# Calcium Transport in the Kidney and Disease Processes

**DOI:** 10.3389/fendo.2021.762130

**Published:** 2022-03-01

**Authors:** Ramy M. Hanna, Rebecca S. Ahdoot, Kamyar Kalantar-Zadeh, Lena Ghobry, Ira Kurtz

**Affiliations:** ^1^ Division of Nephrology, Department of Medicine, University of California Irvine (UCI) School of Medicine, Orange, CA, United States; ^2^ School of Public Health, University of Pittsburgh, Pittsburgh, PA, United States; ^3^ Division of Nephrology, Department of Medicine, David Geffen School of Medicine, University of California Los Angeles (UCLA), Los Angeles, CA, United States; ^4^ University of California Los Angeles (UCLA) Brain Research Center, Los Angeles, CA, United States

**Keywords:** calcium transport, channelopathies, parathyroid signaling, transport physiology, phosphate, signaling

## Abstract

Calcium is a key ion involved in cardiac and skeletal muscle contractility, nerve function, and skeletal structure. Global calcium balance is affected by parathyroid hormone and vitamin D, and calcium is shuttled between the extracellular space and the bone matrix compartment dynamically. The kidney plays an important role in whole-body calcium balance. Abnormalities in the kidney transport proteins alter the renal excretion of calcium. Various hormonal and regulatory pathways have evolved that regulate the renal handling of calcium to maintain the serum calcium within defined limits despite dynamic changes in dietary calcium intake. Dysregulation of renal calcium transport can occur pharmacologically, hormonally, and *via* genetic mutations in key proteins in various nephron segments resulting in several disease processes. This review focuses on the regulation transport of calcium in the nephron. Genetic diseases affecting the renal handling of calcium that can potentially lead to changes in the serum calcium concentration are reviewed.

## Introduction

**Figure 1:**
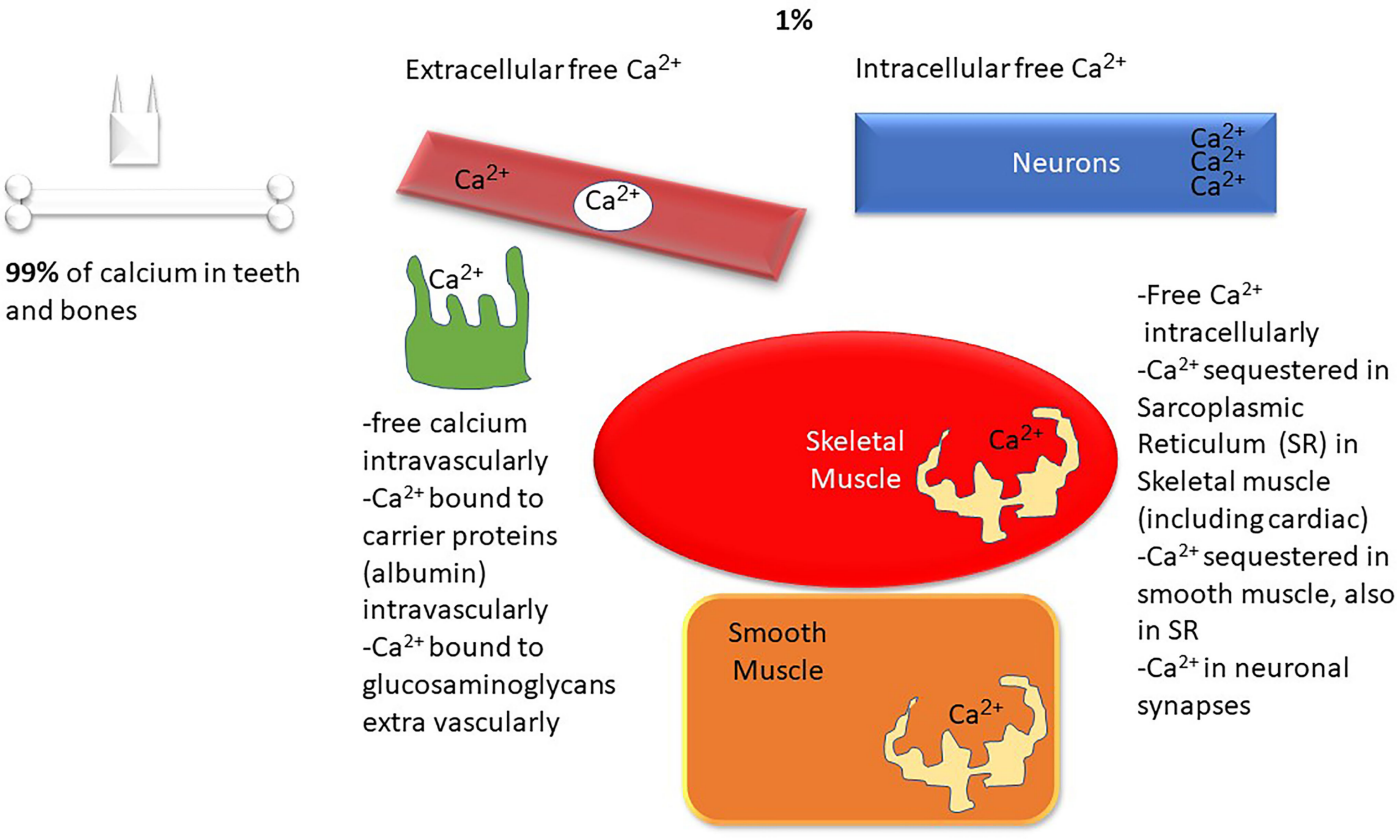
Calcium metabolism overview: Ca^2+^, calcium; SR, sarcoplasmic reticulum.

**Figure 2:**
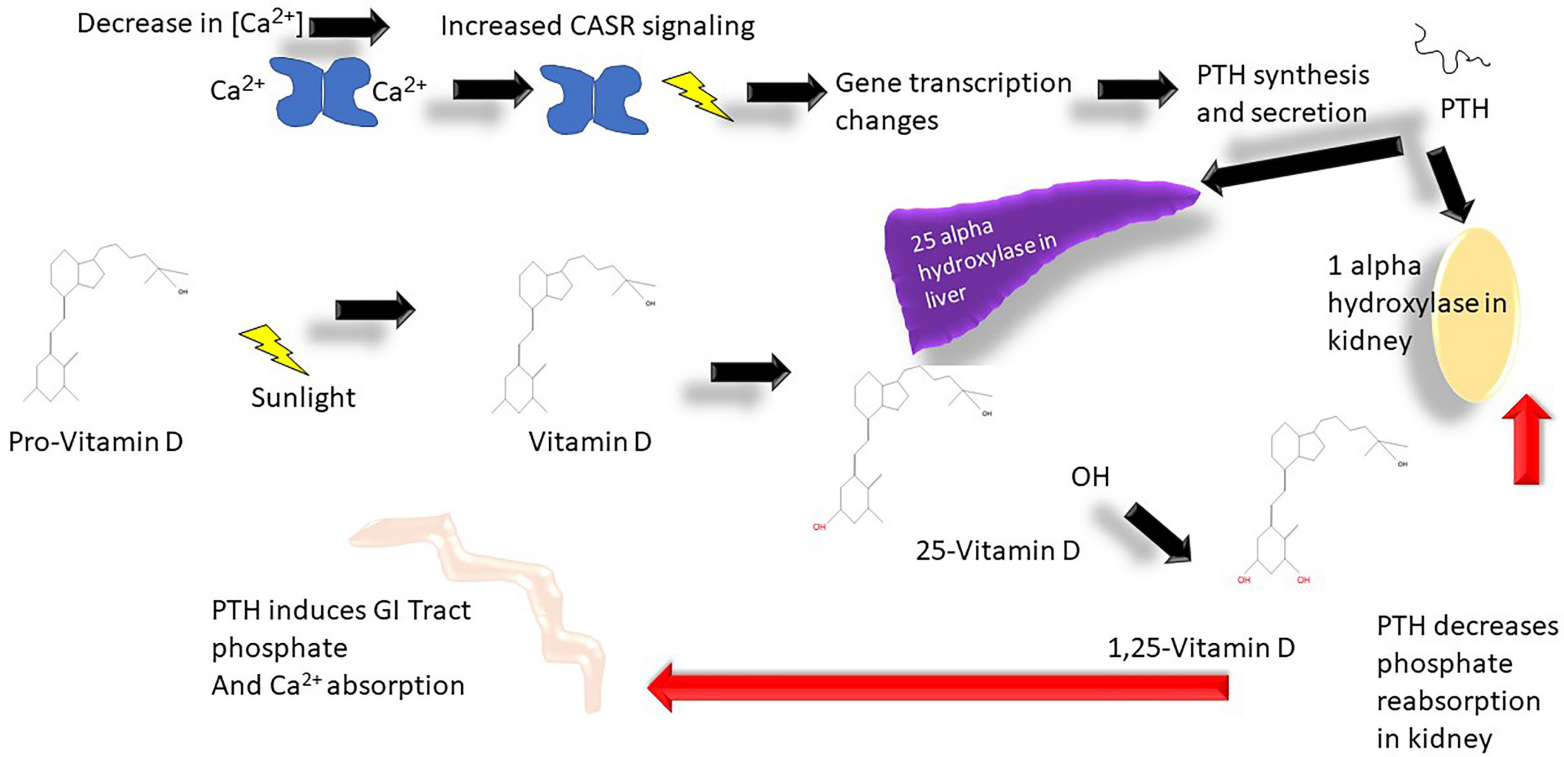
Calcium metabolism vitamin D cycle: Ca^2+^, calcium; CASR, calcium sensing receptor; OH^-^, hydroxyl group.

Calcium is a ubiquitous intracellular and extracellular divalent cation that is involved in structural, biochemical, and metabolic processes throughout the body^1^. Calcium is required for muscle contraction, cardiac contractility, rhythm, normal neurologic function, bone and teeth structure, blood clotting, hormone release, and enzyme function. **Figures 1**, **2** demonstrate the biology of calcium utilization in the body and depict the hormonal regulation of calcium levels in human physiology. In the serum, total calcium (8.9–10.1 mg/dl or 2.2–2.5 mmol/l) is composed of various fractions that are ionized, protein bound (albumin, globulin), and complexed to phosphate and citrate (~ approximately 48%, 45%, and 7%) (1). The intracellular Ca^2+^ is maintained at ~100 nM (similar to the concentration of protons in the cell) and changes dynamically during various intracellular signaling processes (1).

**Figure 3:**
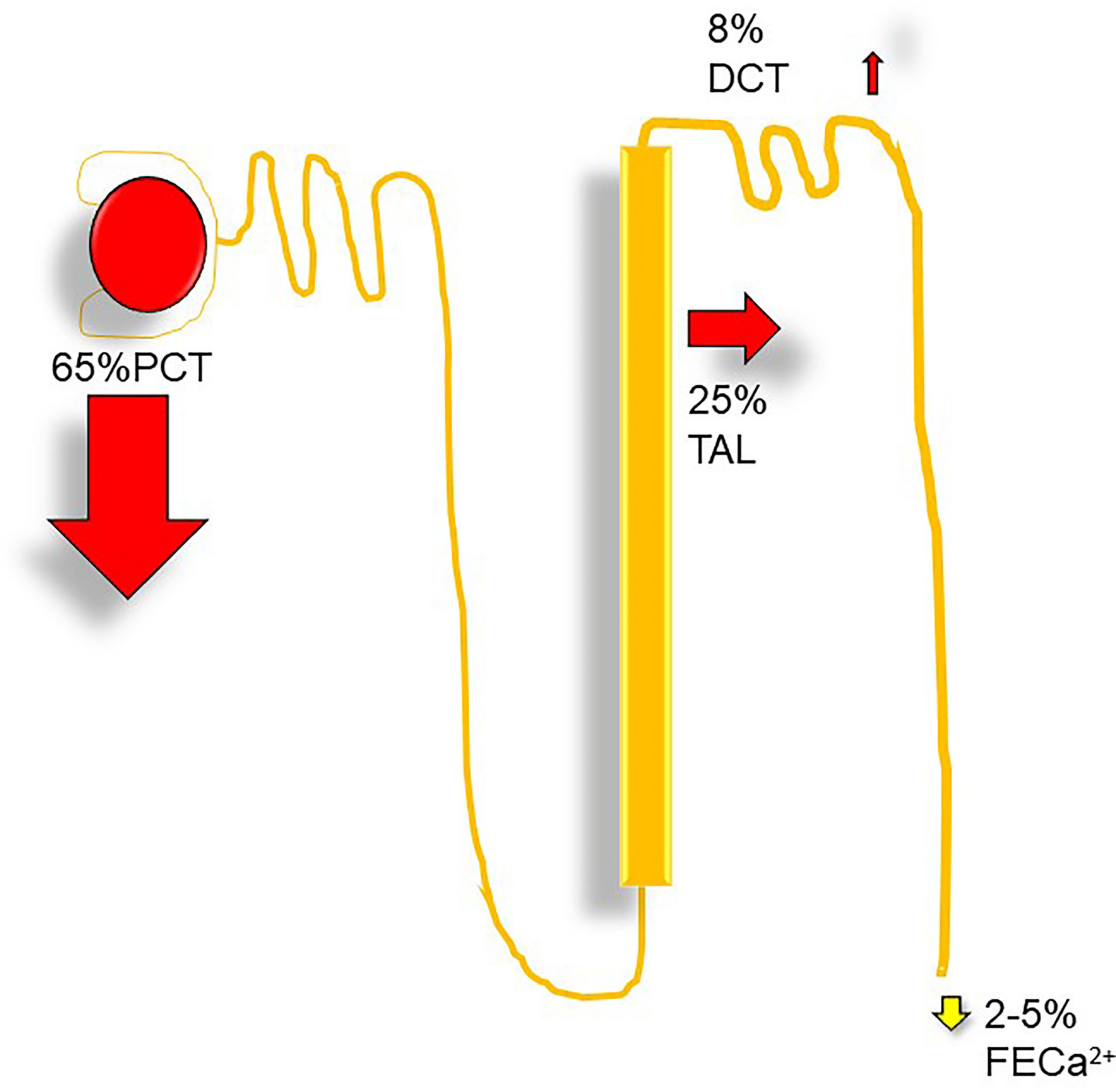
Nephron calcium transport: DCT, distal convoluted tubule, Ca^2+^, fractional excretion of calcium (%); PCT, proximal convoluted tubule; TAL, thick ascending limb.

Filtered calcium represents the ionized and complexed fractions. Per 1.0-g/dl drop in serum albumin, total serum calcium should decline by 0.8 mg/dl (2), and for each 1.0-g/dl decrease in serum globulin, total serum calcium decreases by 0.12 mg/dl (3). With a GFR of ~170 l per 24 h, ~10 g of calcium is filtered (3). 100–200 mg of calcium is normally excreted per day in urine, and about 98% of filtered load is reabsorbed within the nephron. The proximal convoluted tubule reabsorbs 60%–70%, the loop of Henle reabsorbs 20%, the distal convoluted tubule absorbs 10%, and the collecting duct absorbs only 5% (**Figure 3**).

## Renal Calcium Transport

### Proximal Tubule

The reabsorption of calcium within the proximal tubule (PT) mirrors that of sodium and water. In the S1 segment, tubular calcium reabsorption occurs *via* solvent drag and passive diffusion (4). The passive paracellular pathways account for approximately 80% of calcium reabsorption in this segment of the nephron. A small but poorly understood active transcellular calcium transport may also be present in the proximal tubule (4) that can potentially be regulated by parathyroid hormone (PTH) and calcitonin (5). A possible candidate protein that might be involved in transcellular calcium transport transporter is the apical voltage-dependent L-type calcium channel (6). In the S2 proximal tubule segment, passive transcellular calcium transport also occurs due to the generation of a positive lumen voltage of ~ +1 mv as a consequence of Cl^-^ flux down its concentration gradient established in the S1 segment (7).

**Figure 4:**
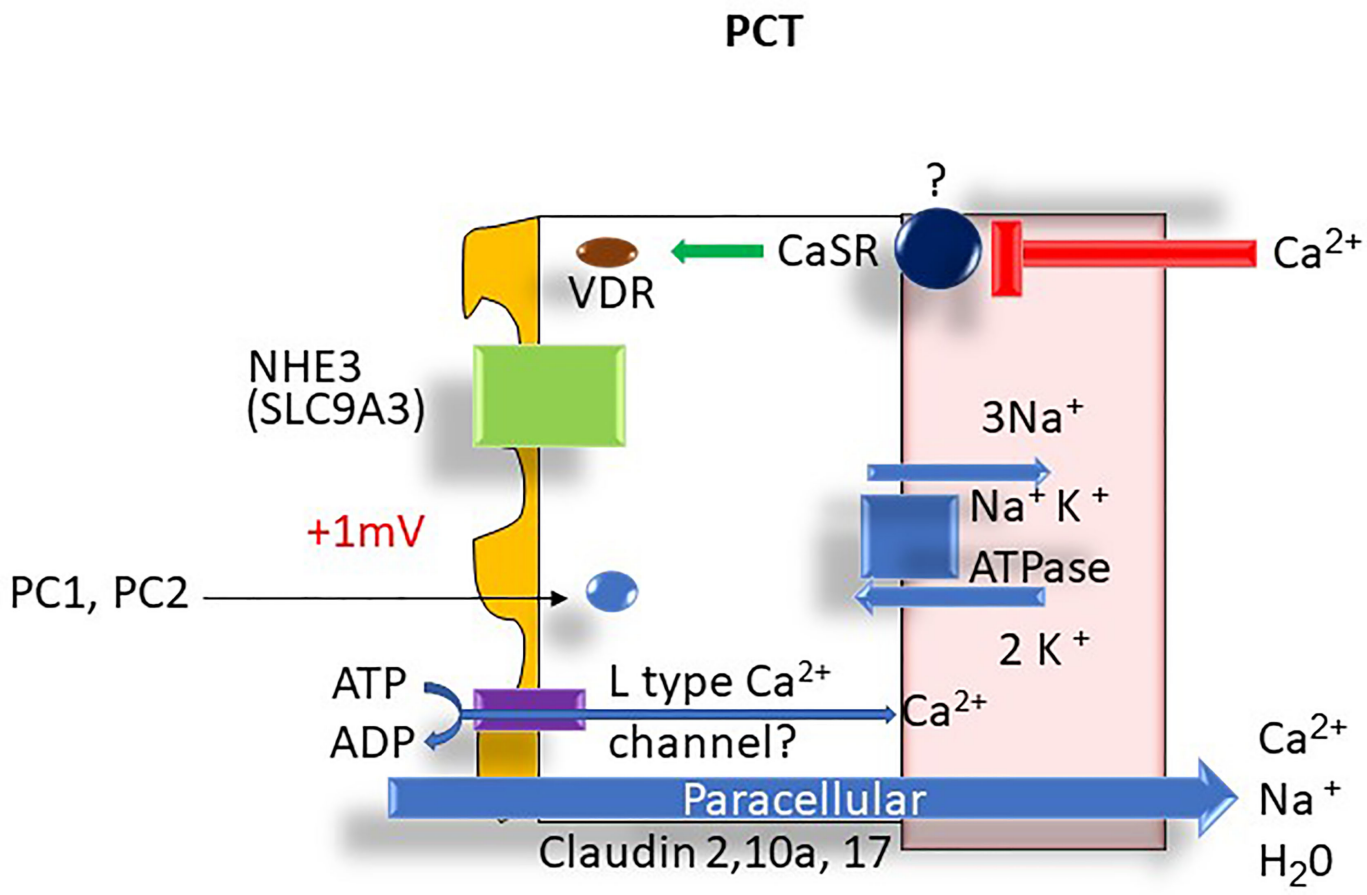
Proximal tubule Ca^2+^ transport: ATPase, adenosine triphosphatase; Ca^2+^, calcium; Cl^-^, chloride; H_2_O, water; K^+^, potassium; mV, millivolt; Na^+^, sodium; NHE3, Na^+^-H^+^ exchanger 3; PCT, proximal convoluted tubule; PC1, polycystin 1, PC2, polycystin 2; VDR, vitamin D receptor.

The main paracellular tight-junction proteins that allow calcium permeation in the PT include the pore-forming Claudins (Claudin 2, 10a, and 17) (5) (see **Figure 4**). Polycystins 1 and 2 (PC1/PC2) are thought to be an important intracellular regulator of intracellular calcium signaling and proximal tubule calcium transport. Members of the transient voltage receptor protein family (8), PC1 and PC2 interact with endoplasmic reticulum (ER) calcium channels resulting in depletion of calcium in the organelle *via* direct transport *via* PC1/PC2 complexes. The depletion of ER calcium then results in activation of store-operated channels (SOC) that then transport calcium into the cytoplasm. This then results in control of cellular proliferation through G protein and MAP kinase regulation resulting in changes to gene regulation. The higher calcium levels also promote vesicle fusion. Mutations in PC1/PC2 result in the manifestations of autosomal dominant polycystic kidney disease 1 and 2 (ADPKD 1,2) (8).

### Ascending Limb

The initial segments of the loop of Henle (thin descending and thin ascending limbs) are relatively calcium impermeable. In the thick ascending limb (TAL), ~20% of the filtered calcium is absorbed largely in the cortical segment (5). The majority of calcium absorption is paracellular like the PT and proportional to the trans-tubular electrochemical driving force (5). The apical Na^+^-K^+^-2Cl^−^ cotransporter, NKCC2, and the renal outer medullary potassium K^+^ (ROMK) channel generate the lumen-positive membrane potential (driving force) for paracellular calcium transport. Although NaCl reabsorption (lumen to cell) through NKCC2 is electroneutral (NKCC2 transports 1 Na^+^, 1 K^+^, and 2 Cl^−^ ions), potassium ions back-diffuse into the lumen through the apical ROMK channels generating a lumen-positive voltage (+10 mv). The basolateral Na^+^-K^+^-ATPase is also involved in maintaining the membrane potential (9). The tight-junction proteins claudin 14, 16, (paracellin) and 19 are thought to play a key role in paracellular calcium flux.

**Figure 5:**
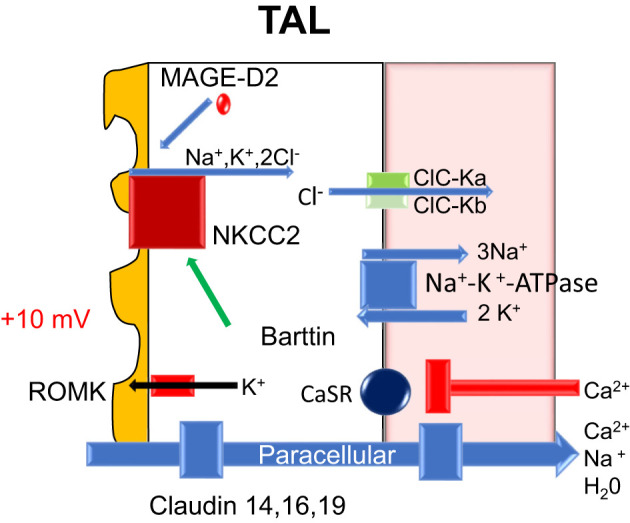
Nephron molecular calcium handling-loop of Henle: ATPase, adenosine triphosphatase; CaSR, calcium sensing receptor; Ca^2+^, calcium; Cl^-^, chloride; H20, water; K^+^, potassium; mV, millivolt; Na^+^, sodium; NKCC2, sodium potassium two chloride transporter; ROMK, apical renal outer medullary potassium channel; TAL, thick ascending of Henle; ClC-Ka, chloride channel kidney A; ClC-Kb, chloride channel kidney B; MAGED-2, melanoma-associated antigen D2.

Calcium transport is also influenced by the basolateral calcium-sensing receptor (CaSR) (10); it is a calcium-binding G protein-coupled receptor found in the thick ascending limb and the parathyroid gland (11). If high calcium levels are detected, signaling through CaSR inhibits the expression of claudin 14, 16 (paracellin), and 19 (12). The lack of available claudin proteins results in inhibition of paracellular calcium reabsorption and hypercalciuria (12) (see **Figure 5**).

In the *in vitro* perfused rat cortical TAL, an acute inhibition of CaSR increased paracellular calcium permeability but did not alter NaCl reabsorption or the transepithelial potential difference. Toka et al. (13) noted that CaSR disruption decreases the abundance of claudin-14 mRNA and claudin-16 mRNA (14). Cinacalcet increased the abundance of claudin-14 mRNA, and in cell culture models overexpression of claudin-14 decreased the paracellular permeability to calcium (5). Calciotropic hormones, such as PTH and calcitonin, stimulate calcium absorption in the cortical thick ascending limb (15) (**Figure 5**).

### Distal Convoluted Tubule

**Figure 6:**
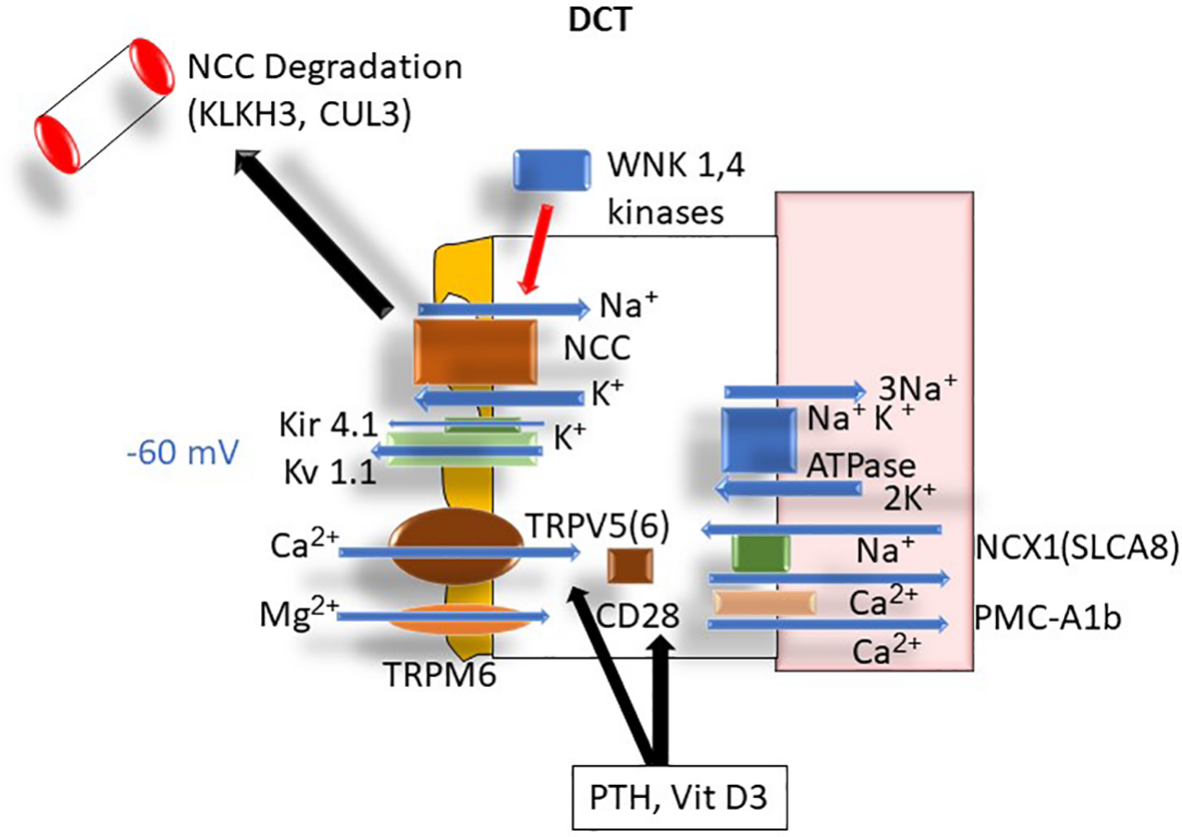
DCT Ca^2+^ transport: ATPase, adenosine triphosphatase; Ca^2+^, calcium; CD28, cellular determinant 28 (Calbindin); CUL3, cullin 3; DCT, distal convoluted tubule; K^+^, potassium; Kir 4.1, inwardly rectifying potassium channel; KLHL3, Kelch-like protein 3*;* Kv 1.1, apical potassium channel 1.1; Na^+^, sodium; NCC, thiazide-sensitive sodium channel; NCX-1, sodium-calcium exchanger-1 (aka SLCA8); PMCA1b, plasma membrane calcium adenosine triphosphatase (ATPase); TRPM6, transient receptor protein magnesium channel 6; WNK 1,4, lysine-deficient protein kinase 1.4.

In contrast to PT and TAL, in the distal convoluted tubule (DCT) calcium is absorbed transcellularly *via* the transient receptor potential cation channel subfamily V member 5 (TRPV5) and TRPV6 channels on the apical membrane (16) where TRPV5 is the major Ca^2+^ channel involved in Ca^2+^ influx (16). Luminal potassium extrusion *via* the apical Kv 1.1 channel plays an important role in determining the apical membrane voltage (17). Interestingly, membrane depolarization has not been reported to affect the TRPV5 activity whereas hyperpolarization increases TRPV5 activity, promoting Ca^2+^ uptake into the cells (18). In the cytoplasm, calbindin-D28k binds intracellular calcium and shuttles it through the cytosol toward the basolateral membrane. Basolateral calcium extrusion is mediated by the sodium-calcium exchanger-1 (NCX1; SLC8A1) (19) and plasma membrane Ca^2+^-ATPase PMCA1b (20). Via changes in apical and basolateral membrane voltages and the intracellular Na^+^ concentration, DCT handling of calcium is modulated by the activity of the apical thiazide sensitive sodium chloride cotransporter (NCC), WNK kinases (alters NCC activity), basolateral Kir4.1/5.1 K^+^ channels (alters the intracellular Cl^-^ concentration), and basolateral ClC-Kb Cl^-^ channels (20) (**Figure 6**).

### Cortical Collecting Duct

**Figure 7:**
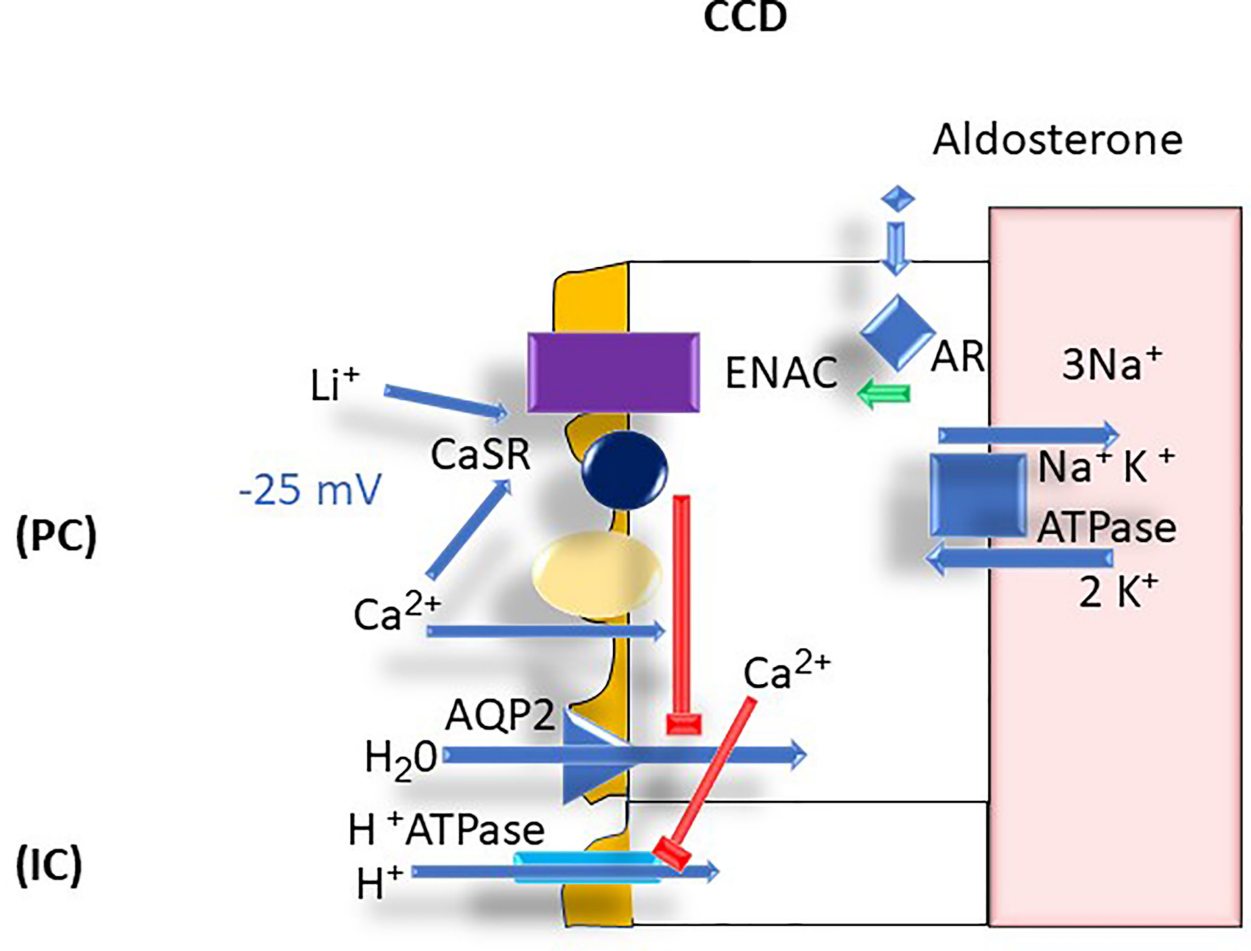
CCD Ca^2+^ transport: AR, aldosterone receptor; AQP2, aquaporin 2; ATPase, adenosine triphosphatase; Ca^2+^, calcium; CaSR, calcium sensing receptor; CCD, cortical collecting duct, ENaC, epithelial sodium channel; H^+^, proton; H_2_O, water; IC, intercalated cells; K^+^, potassium; Li^+^; lithium; mV, millivolt; Na^+^; sodium; PC, principal cells.

Calcium plays an important physiologic function in the CCD in that it inhibits apical aquaporin 2 (AQP2) expression (21). The presence of CaSR on the cortical collecting duct cells has been proposed to be the involved mechanism but is not confirmed (22). The inhibition of AQP2 can readily explain the polyuria that results from hypercalcemia with associated hypercalciuria (22). Calcium is also thought to stimulate luminal H^+^ secretion *via* the type A intercalated cell apical H^+^-ATPase resulting in the excretion of a more acidic urine (23). These homeostatic mechanisms are thought to prevent stone formation by diluting the luminal calcium concentration and acidifying the urine, thereby enhancing calcium phosphate solubility (24). However, polyuria per se can be counterproductive in that it leads to dehydration and potentially hypernatremia given insufficient water intake (24) (**Figure 7**).

### Regulation of Calcium Transport in Nephron

#### PTH and Vitamin D

PTH that is secreted by the parathyroid gland in response to variations of serum Ca^2+^ results in changes in intestinal absorption of calcium *via* enhanced 1,25(OH)_2_ D (D3) production and changes in DCT renal calcium absorption. The regulation of PTH is intimately controlled by calcium concentration and occurs at the level of transcription and changes in intracellular degradation of PTH (25). PTH release is triggered by hypocalcemia and modulated by prostaglandin E2, dopamine, and adrenergic agonists (25). In the parathyroid gland Ca^2+^ is sensed by the CaSR which controls PTH secretion (26). CaSR induces through cyclic AMP transcription of parathyroid hormone (PTH) (26). FGF23 receptor (Klotho) expressed on the parathyroid gland also regulates PTH secretion (27). Klotho is stimulated by hyperphosphatemia to positively regulate the secretion of PTH secretion. If the serum calcium concentration drops this results in increased PTH secretion, which induces 1-alpha-hydroxyalse transcription in the kidneys and promotes 25-alpha-hydroxylase in the liver (28) (**Figures 2**, **6**).

In the DCT, PTH and vitamin D are involved specifically also in regulation of calcium at the transport level through regulation of calbindin (intracellular protein) and TRPV5 and TRPV6 as well as CD28/calbindin. Depletion of vitamin D results in decreased expression whereas both high levels of vitamin D and PTH result in higher expression of TRPV5 and 6 and CD28 (1). Once PTH is secreted, the effect of increasing vitamin D3 production also stimulates calcium and phosphate absorption through the GI tract. Vitamin D3 then enters the enterocyte resulting in binding to the intracellular vitamin D receptor, and expression of various proteins. Calcium transport can occur *via* channels, active transport, and paracellular transport across enterocytes—and these processes are regulated by vitamin D3. The specific proteins include calbindin, PMCA3 (mediator complex subunit Med27), and the exchanger NCX1 expressed in the luminal surface of enterocytes. This is in addition to channels like TRPV6, and paracellular transport *via* claudins 2, 12, and 15 across the enterocyte (29) (see **Figure 2**). There are other proteins active in calcium transport in the enterocyte: calcium channel, voltage-dependent, L type, and alpha 1D subunit (Cav 1.3) are involved as well in calcium entry into enterocytes; active transport is mediated by the plasma membrane Ca^2+^-ATPase (PMCA1b), and finally Calbindin9k allows increased enterocyte calcium absorption in response to vitamin D3 (**Supplemental Figure A**).

#### Serum Calcium

Changes in urinary calcium excretion can occur secondary to an alteration on the blood calcium concentration with concomitant changes in the filtered load rate and rate of tubular calcium absorption. Hypercalcemia results with increased urine calcium excretion, due to a higher filtered load and lower rate of calcium reabsorption by the nephron (30). Hypercalcemia can cause renal vasoconstriction which tends to lower the filtered load (30). Renal calcium excretion drops in hypocalcemia mainly through the mechanism of a lower filtered load and a compensatory increase in tubular calcium reabsorption (30).

#### Acid Base

Alterations in urine pH can result in hypercalciuria in the DCT segment; chronic metabolic acidosis results in hypercalciuria, whereas alkalinization results in decreased urinary calcium excretion (1). The urine calcium excretion varies with the serum bicarbonate concentration (31). This is known to be due to changes in renal tubular calcium absorption rather than changes in the filtered load (32–34). The likely mechanism is the pH effects on the TRPV5 calcium channel in the DCT (18, 35, 36).

#### Extracellular Fluid Volume

Volume expansion decreases tubular absorption of sodium, chloride, and calcium with opposite changes occurring during volume contraction (29). The major site of this effect is thought to be the proximal tubule (29). During volume expansion, there is an increase in GFR and filtered load of calcium (4, 37). The decrease in proximal tubular calcium absorption is proportional to the decrease in sodium and water absorption such that the luminal calcium concentration remains unchanged (29). The absolute amount of calcium absorbed in the loop of Henle during volume expansion is increased above control (29).

#### Diuretics

In the proximal tubule, osmotic diuretics (i.e., mannitol) block paracellular water reabsorption from the decreased calcium absorption through a solvent drag mechanism (38). Acetazolamide also blocks calcium absorption by blocking water absorption. This effect is mediated by the creation of a luminal disequilibrium pH that inhibits bicarbonate absorption mediated by NHE3 (39). SGLT2 inhibitors block sodium-glucose co-transport (and possibly NHE3) in the proximal tubule, resulting in a glucose-induced osmotic diuresis and impaired bicarbonate transport (40). In a similar fashion, calcium absorption is impaired.

In the TAL, diuretics decrease calcium absorption by competing for the chloride site on the Na-K-2Cl cotransporter (41). Inhibiting NKCC2 sodium chloride reabsorption inhibits the back leak of potassium *via* ROMK and impairs the generation of the lumen-positive potential needed to drive paracellular calcium absorption. In neonates, chronic use of loop diuretics can be deleterious leading to the development of nephrocalcinosis (42).

In the DCT and connecting tubule, the direct effect of thiazides in the DCT is to decrease NCC transport and calcium absorption (43, 44). However, in the whole kidney, thiazide diuretics significantly decrease renal calcium excretion due to enhanced proximal tubule calcium absorption as a result of hypovolemia (44). There are accompanying changes in distal calcium delivery that modulate calcium transport in the DCT (45, 46). Postulated mechanisms for this effect include increased entry of luminal calcium *via* TRPV5, enhanced basolateral extrusion *via* the Na-Ca exchanger, and decreased levels of calcium transporters (47, 48). If thiazide therapy increases plasma calcium above 12 mg/dl or if hypercalcemia persists, primary hyperparathyroidism or another hypercalcemic state should be suspected (49).

In the cortical collecting duct, amiloride increases calcium reabsorption and reduces calcium excretion (38, 50). The mechanism by which amiloride reduces calcium excretion is not well understood. The effect of amiloride may involve both the connecting tubule in which sodium entry occurs *via* both ENaC Na^+^ channels and NCC cotransporters (46) and the initial cortical collecting tubule where cellular Na^+^ entry occurs only *via* ENaC (46, 51, 52). By hyperpolarizing the apical membrane amiloride promotes calcium influx *via* TRPV5 channels (50). Little is known of the effect of spironolactone and eplerenone on urinary calcium excretion in normal subjects. Spironolactone or adrenalectomy can reduce hypercalciuria (53).

#### FGF23

FGF23 plays an extensive role in renal phosphate handling (5, 27, 54, 55). FGF23 secreted in response to hyperphosphatemia binds to FGF23R/Klotho receptors in the parathyroid and the PCT. The downstream signaling effects are achieved *via* calcineurin (CN) and mitogen-activated protein -kinase (MAP-K) pathways (27), due to the regulation of 1-alpha-hydroxylase and PTH by FGF23 (54). FGF23 exerts its effects *via* mitogen-activated kinase (MAPK) signaling, influencing the intracellular sodium hydrogen exchange regulatory factor-1 (NHERF-1) (55).

The actions of phosphatonins on calcium are intertwined with FGF23’s regulatory action on vitamin D metabolism (27, 56). In the PCT, FGF23 decreases 1-alpha-hydroxylase thereby decreasing the level of active vitamin D3. It also increases 24-hydroxylase which results in active D_3_ hydroxylation and deactivation (27, 56). In doing so, FGF23 results in decreased vitamin D signaling with the vitamin D receptor (27, 56). This results in decreased calcium absorption systemically but does not result in hypocalcemia (27, 56). FGF23 also results in increased PTH signaling form the parathyroid gland. In aggregate, FGF23 stimulates PTH-mediated effects on phosphate secretion while inhibiting vitamin D3 production (27, 56) (**Supplemental Figure A**).

PTH and FGF23 modulate phosphate reabsorption through inhibition of apical sodium-phosphate (NaPi) cotransporters which reabsorb phosphate (28). They do so through protein kinase A and C regulation. Specifically, the apical transporters NaPi2a, NaPi2c, and Pit-2 are endocytosed resulting in decreased function of these phosphate transporters in the PCT brush border (5) (see **Supplemental Figure A**).

#### CKD

In patients with chronic kidney disease (CKD), FGF23 levels rise spontaneously in response to hyperphosphatemia (26). Secondary hyperparathyroidism occurs due to rising uremic toxins and increasing phosphate (26); an effect that is CKD stage dependent (26). There is associated decreased renal calcium excretion and decreased intestinal calcium absorption. In end-stage renal disease (ESRD), these effects are more pronounced (27).

### Genetic Disorders of Renal Calcium Transport and Clinical Syndromes

#### Vitamin D and Vitamin D Receptor

Vitamin D-resistant ricket disorders are caused by mutations in vitamin D synthesis and cytochrome P450 proteins (57). One such mutation is in 1-alpha hydroxylase (CYP27B1-chromosome 12) that is the liver enzyme for production of 1-hydroxy-vitamin D_3_. Hypocalcemia and hypophosphatemia may sometimes be present. Elevated PTH and alkaline phosphatase levels are present seen due to high levels of skeletal turnover are usually seen. This autosomal recessive disorder results in a phenotype of osteomalacia, short stature, dental caries, and genu varum.

Another cause of vitamin D-resistant rickets is due to vitamin D receptor mutations encoded on chromosome 12q13.11. The inheritance pattern is autosomal recessive, and the biochemistry profile is similar to other cases of vitamin D-resistant rickets (58). X-linked hypophosphatemic rickets is encoded in the PHEX gene and does not affect calcium metabolism (59).

#### CaSR Mutations

Disorders resulting in low serum calcium include autosomal dominant hypocalcemia (types type 1 and 2) (60). Type 1 is typically caused by mutations in CaSR, where a constitutively active CaSR results in decreased levels of PTH and vitamin D3. Constitutive activation of CaSR (type 1; autosomal dominant) results in reduced calcium absorption and hypocalcemia (60). Type 2 autosomal dominant hypocalcemia is typically caused by mutations in the GNA11 gene (encoded in chromosome 19p13.3), which produces G protein alpha subunit 11 that regulates CaSR (61). Hypocalcemia is due to decreased intestinal calcium absorption. FHH (familial hypercalcemia with hypocalciuria) results from inactivating mutations in CaSR. This disorder results in a higher-than-normal constitutive expression of PTH resulting in a mimic of hyperparathyroidism. Clinically, a modest elevation of serum calcium and PTH are present associated with an abnormally low urine calcium excretion rate, as opposed to the normal or high rate of calcium excretion in primary hyperparathyroidism (60). In cases of 2 nonfunctional CaSR alleles or mutations that result in severely reduced CaSR activity, the phenotype is more severe and is referred to as neonatal severe hyperparathyroidism.

#### PTH Mutations

Familial hypoparathyroidism (autosomal recessive) can result from parathyroid hormone loss-of-function mutations (encoded on chromosome 3p21) which can also occur resulting in poor or absent hormonal signaling (62). Predictably, inactivating mutations result in poor GI calcium absorption and hypocalcemia. Genetic syndromes of magnesium wasting (TRPM6, SLCA4 mutations) can mimic inactivating PTH mutations, because PTH is unable to function properly without magnesium as a cofactor (62).

Familial hyperparathyroidism (autosomal dominant) can conversely result from gain-of-function mutations in PTH, encoded as above (63). The clinical presentation is associated with high levels of serum calcium and low levels of serum phosphate without increased PTH. Hypercalciuria is expected in familial hyperparathyroidism cases, in contrast to FHH (63).

#### PTH-Resistant Hypoparathyroidism

Patients with PTH-resistant hypocalcemia may have several mutations in transcription factor proteins including glial cells missing protein (GCM2), T box-1 mutations (TBX-1), SRY Box 3 (SOX3), GATA-binding protein 3 (GATA3), and tubulin-specific chaperone E. These mutations confer a resistance to PTH and vitamin D3 by affecting vitamin D3 receptor and PTH receptor expression. Transmission is autosomal recessive, and clinically the phenotype is severe hypocalcemia with high PTH levels (PTH resistance) (11).

#### Multiple Endocrine Neoplasia

These disorders result from mutations in MEN1 and CDC73/HPRT2 genes which map to chromosome 11q13 (64). These diseases are transmitted in an autosomal dominant manner and clinically present with hypercalcemia from parathyroid malignancies overproducing PTH. They differ from FHH based on the clinical pattern of urinary hypocalciuria (as opposed to normal or hypercalciuria in MEN1) and the risk of endocrine malignancy with MEN1 mutations.

#### William’s Syndrome

William’s syndrome (autosomal dominant) is another genetic cause of hypercalcemia. It is caused by mutations in elastin and actin binding (LIM) kinase. Both of these proteins have been localized to genes in 7q11.23 (65). The phenotype of William’s syndrome includes hypercalcemia and behavioral abnormalities with a diminished fear response and loss of caution when approaching strangers (66).

#### Bartter’s Syndrome

Bartter’s syndrome causes hypercalciuria and a hypokalemic metabolic alkalosis without a change in the serum calcium. There are various types of Barrter’s syndrome (Types 1-6) involving mutations in genes encoding NKCC2, ROMK, ClC-Ka, ClC-Kb, Barttin, CaSR, and MAG-D2 (67). The effect of loop diuretics on the TAL often mimics the findings in these disorders (67–69).

#### Gitelman’s Syndrome

Gitelman’s syndrome, an autosomal recessive disorder, is caused by mutations in NCC in the DCT. Like Bartter’s syndrome, patients with Gitelman’s syndrome have metabolic alkalosis and hypokalemia. Unlike most types of Bartter's syndrome, patients have hypocalciuria and their phenotype mimics the action of thiazide diuretics (70). Decreased renal calcium excretion is thought to be due to enhanced proximal tubule calcium absorption as a result of hypovolemia (44) and possibly *via* enhanced calcium reabsorption distally at the thiazide-sensitive site in the distal tubule and connecting segment (45, 46).

#### Gordon’s Syndrome

Gordon’s syndrome is an autosomal dominant disorder that clinically presents with hypertension, hyperkalemia, and hypercalciuria (71). The four most common mutations are WNT-signal transduction kinase 1 (WNK1), WNT-signal transduction kinase 4 (WNK4), Kelch-like protein 3 (KLHL3), and Cullin-3 (CUL3) (71). WNK1 and WNK 4 are kinases that negatively regulate the NCC transporter. KLHL3 and CUL3 are components of the ubiquitin degradation proteosome which degrade the WNK1 and 4 protein products (71). Loss-of-function mutations result in increased sodium reabsorption *via* NCC activity, decreased potassium excretion, hypertension, and hyperkalemia. Renal calcium excretion can be increased in Gordon’s syndrome and may lead to nephrocalcinosis (71).

#### EAST/SESAME Syndrome

EAST syndrome is an abbreviation for a clinical syndrome of epilepsy, ataxia, sensorineural deafness, and tubulopathy (72). The syndrome is also called SESAME syndrome (seizures, sensorineural deafness, ataxia, mental disability, and electrolyte imbalance) (73). Inactivating mutations in the basolateral inward rectifying Kir 4.1 potassium channel in the DCT cause the syndrome (72). Kir 4.1 is also expressed in neuronal tissue accounting for the complex phenotype. Patients present with hypokalemia, metabolic alkalosis, hypomagnesemia, and hypocalciuria. See **Supplemental Table A**.

#### Claudin Mutations

Mutations in claudin 16 and 19 (familial hypomagnesemia with hypercalciuria and nephrocalcinosis, FHHNC), result in hypercalciuria, nephrocalcinosis, nephrolithiasis and renal failure (74). See **Table 1**.

**Table 1:** Genetics of calcium metabolic disorders.

Mutation type	Chromosome location
Autosomal dominant hypocalcemia-type 1 CaSR activation mutation autosomal dominant	Chromosome 3.122.18
Autosomal dominant hypocalcemia-type 2 GNA11 mutations autosomal dominant	Chromosome 19p13.3
Familial PTH-resistant hypoparathyroidism-GCM-2 autosomal recessive	Chromosome 16.10.87
Familial PTH-resistant hypoparathyroidism-TBX-1 autosomal recessive	Chromosome 22.11.21
Familial PTH-resistant hypoparathyroidism-SOX3 X-linked recessive	X Chromosome 140.5
Familial PTH-resistant hypoparathyroidism-GATA3 autosomal recessive	Chromosome 10p14
Familial PTH-resistant hypoparathyroidism-tubulin-specific chaperone E autosomal recessive	Chromosome 13
Vitamin D-resistant rickets 1-alpha hydroxylase (CYB27A1) autosomal recessive	Chromosome 12
Vitamin D-resistant rickets-vitamin D receptor autosomal recessive	Chromosome 12q13.11
Hypomagnesemia with secondary hypocalcemia (HSH)-TRMP6 autosomal recessive	Chromosome 9q21.13
Hypomagnesemia with secondary hypocalcemia (HSH)-SLC4A1 autosomal recessive	Chromosome 17q21-22
Familial hypoparathyroidism-PTH-inactivating mutations autosomal recessive	Chromosome 11q13
Familial hypoparathyroidism: PTH-activating mutations autosomal dominant	Chromosome 11q13
MEN 1-hypercalcemia and malignancy due to parathyroid malignancy autosomal dominant	Chromosome 7q11.23
MEN1- CDC 73/HRPT mutations autosomal dominant	Chromosome 11q13
FHH- CaSR-inactivating mutation autosomal recessive (less severe mutation)	Chromosome 3.122.18
Neonatal severe hyperparathyroidism-CaSR more severe mutation	Chromosome 3.122.18
Williams syndrome-elastin Autosomal dominant	Chromosome 7q11.23
Williams syndrome-actin-binding [LIM] kinase Autosomal dominant	Chromosome 7q11.23
FHHNC - claudin 16 autosomal recessive	Chromosome 3q27
FHHNC - claudin 19 autosomal recessive	Chromosome 1p34.2
Gordon’s syndrome I WNK-1 autosomal dominant	Chromosome 12p13.33
Gordon’s syndrome II WNK-4 autosomal dominant	Chromosome 17q21-22
Gordon’s syndrome III KLHL-3 autosomal dominant	Chromosome 5q31
Gordon’s syndrome IV CUL3 autosomal dominant	Chromosome 2q36
Bartter’s syndrome I- NKCC2 (SLC12A1) autosomal recessive	Chromosome 18q12.1
Bartter’s syndrome II- ROMK or (KCNJ1) autosomal recessive	Chromosome 11q24
Bartter’s syndrome III-Barttin autosomal recessive	Chromosome 1.16.04
Bartter’s syndrome IV-sodium/K ATPase type IV subunits autosomal recessive	Chromosome 1.16.04
Bartter’s syndrome V- severe activating CaSR activations, autosomal dominant MAGE-D2 Type VI Neonatal transient Bartter’s syndrome, chromosome mapping, and X linked dominant transmission	Chromosome 3.122.18
Gitelman’s syndrome-NCC or (SLC12A3) autosomal recessive	Chromosome 16q13
EAST/SESAME syndrome (KCNJ10) [Kir 4.1] autosomal recessive	Chromosome 1q23.2

CaSR, calcium-sensing receptor; EAST, epilepsy, ataxia, sensorineural deafness, tubulopathy; FHH, familial hypercalcemia with hypocalciuria; FHHNC, familial primary hypomagnesemia with hypercalciuria and nephrocalcinosis; GATA 3, GATA-binding protein 3; GCM2, glial cells missing 2; GNA11, gene producing G protein 11; HRPT, hypoxanthine phosphoribosyltransferase; HSH, hypomagnesemia with secondary hypocalcemia; MEN, multiple endocrine neoplasia; NCC, thiazide-sensitive cotransporter; NKCC2, sodium potassium two chloride transporter; PTH, parathyroid hormone; ROMK, renal outer medulla potassium channel; SESAME syndrome (seizures, sensorineural deafness, ataxia, mental retardation, and electrolyte imbalance); SLC4A1, band 3 anion transport protein; SOX3, SRY Box 3; TBX-1, T box 1 mutations; TRPM6, TRP magnesium-permeable channel 6; WNK 1, lysine-deficient protein kinase 1; WNK 4, lysine-deficient protein kinase 4 [note chromosome mapping convention q, long arm; p, short arm].

## Summary

We present here an overview of normal renal calcium handling and a systematic summary of the regulation of renal calcium transport in the nephron. Dysregulation of renal calcium transport can occur pharmacologically, hormonally and *via* genetic mutations in key proteins in specific nephron segments resulting in various diseases processes.

## Author Contributions

RH, RA, KK-Z, and IK wrote the manuscript. LG assisted with the figures. RH and IK edited the final manuscript. All authors contributed to the article and approved the submitted version.

## Funding

IK is supported in part by funds from the NIH (R01-DK077162), the Allan Smidt Charitable Fund, the Factor Family Foundation, and the Ralph Block Family Foundation. KK-Z is supported by the National Institute on Aging of the National Institutes of Health grant R21-AG047036 and the National Institute of Diabetes, Digestive and Kidney Disease grants R01-DK078106, R01-DK096920, U01-DK102163, and K24-DK091419, as well as philanthropist grants from Mr. Harold Simmons and Mr. Louis Chang.

## Conflict of Interest

The authors declare that the research was conducted in the absence of any commercial or financial relationships that could be construed as a potential conflict of interest.

## Publisher’s Note

All claims expressed in this article are solely those of the authors and do not necessarily represent those of their affiliated organizations, or those of the publisher, the editors and the reviewers. Any product that may be evaluated in this article, or claim that may be made by its manufacturer, is not guaranteed or endorsed by the publisher.

## Supplementary Material

Supplemental Figure A Nephron Phosphate Handling: CNI, calcineurin pathway; FGF23, fibroblast growth factor 23; FGF23R, fibroblast growth factor receptor; HPO_4_^2-^, H_2_PO_4_^-^, organic phosphate anions; MAPK, map kinase; NHERF-1, Na^+^/H^+^ exchanger regulatory factor 1; Na K ATPase, sodium potassium ATPase; NaPi2a, sodium phosphate cotransporter 2a; NaPi2c, sodium phosphate cotransporter 2c; PiT2, phosphate transporter; PKA/PKC, protein kinase A/C; PTH, parathyroid hormone.

## References

[1] Jeon US. Kidney and Calcium Homeostasis. Electrolyte Blood Press (2008) 6:68–76. doi: 10.5049/EBP.2008.6.2.68 24459525 PMC3894479

[2] Lian IA Asberg A. Should Total Calcium be Adjusted for Albumin? A Retrospective Observational Study of Laboratory Data From Central Norway. BMJ Open (2018) 8:e017703. doi: 10.1136/bmjopen-2017-017703 PMC5892769 29627804

[3] Besarab A Caro JF. Increased Absolute Calcium Binding to Albumin in Hypoalbuminaemia. J Clin Pathol (1981) 34:1368–74. doi: 10.1136/jcp.34.12.1368 PMC494605 7328184

[4] Suki WN. Calcium Transport in the Nephron. Am J Physiol (1979) 237:F1–6. doi: 10.1152/ajprenal.1979.237.1.F1 380361

[5] Blaine J Chonchol M Levi M. Renal Control of Calcium, Phosphate, and Magnesium Homeostasis. Clin J Am Soc Nephrol (2015) 10:1257–72. doi: 10.2215/CJN.09750913 PMC4491294 25287933

[6] Satin J Schroder EA Crump SM. L-Type Calcium Channel Auto-Regulation of Transcription. Cell Calcium (2011) 49:306–13. doi: 10.1016/j.ceca.2011.01.001 PMC3097264 21295347

[7] Kurtz I. NBCe1 as a Model Carrier for Understanding the Structure-Function Properties of Na(+) -Coupled SLC4 Transporters in Health and Disease. Pflugers Arch (2014) 466:1501–16. doi: 10.1007/s00424-014-1448-8 PMC4096079 24515290

[8] Brill AL Ehrlich BE. Polycystin 2: A Calcium Channel, Channel Partner, and Regulator of Calcium Homeostasis in ADPKD. Cell Signal (2020) 66:109490. doi: 10.1016/j.cellsig.2019.109490 31805375 PMC6935422

[9] Suki WN Rouse D Ng RC Kokko JP. Calcium Transport in the Thick Ascending Limb of Henle. Heterogeneity of Function in the Medullary and Cortical Segments. J Clin Invest (1980) 66:1004–9. doi: 10.1172/JCI109928 PMC371537 7430341

[10] Desfleurs E Wittner M Simeone S Pajaud S Moine G Rajerison R. Calcium-Sensing Receptor: Regulation of Electrolyte Transport in the Thick Ascending Limb of Henle's Loop. Kidney Blood Press Res (1998) 21:401–12. doi: 10.1159/000025892 9933824

[11] Bastepe M. A Gain-Of-Function CASR Mutation Causing Hypocalcemia in a Recessive Manner. J Clin Endocrinol Metab (2018) 103:3514–5. doi: 10.1210/jc.2018-01340 PMC6669810 30020481

[12] Vezzoli G Terranegra A Rainone F Arcidiacono T Cozzolino M Aloia A. Calcium-Sensing Receptor and Calcium Kidney Stones. J Transl Med (2011) 9:201. doi: 10.1186/1479-5876-9-201 22107799 PMC3339356

[13] Loupy A Ramakrishnan SK Wootla B Chambrey R de la Faille R Bourgeois S. PTH-Independent Regulation of Blood Calcium Concentration by the Calcium-Sensing Receptor. J Clin Invest (2012) 122:3355–67. doi: 10.1172/JCI57407 PMC3428075 22886306

[14] Toka HR Al-Romaih K Koshy JM DiBartolo S 3rd Kos CH Quinn SJ. Deficiency of the Calcium-Sensing Receptor in the Kidney Causes Parathyroid Hormone-Independent Hypocalciuria. J Am Soc Nephrol (2012) 23:1879–90. doi: 10.1681/ASN.2012030323 PMC3482734 22997254

[15] Felsenfeld A Rodriguez M Levine B. New Insights in Regulation of Calcium Homeostasis. Curr Opin Nephrol Hypertens (2013) 22:371–6. doi: 10.1097/MNH.0b013e328362141e 23736839

[16] Peng JB Suzuki Y Gyimesi G Hediger MA. TRPV5 and TRPV6 Calcium-Selective Channels. In: Kozak JA Putney JW Jr., editors. In Calcium Entry Channels in Non-Excitable Cells. Chapter 13. Boca Raton (FL): CRC Press/Taylor & Francis (2018). p. 241–74. 30299660

[17] Ellison DH. The Voltage-Gated K+ Channel Subunit Kv1.1 Links Kidney and Brain. J Clin Invest (2009) 119:763–6. doi: 10.1172/JCI38835 PMC2662576 19348045

[18] Hoenderop JG van der Kemp AW Hartog A van Os CH Willems PH Bindels RJ. The Epithelial Calcium Channel, ECaC, Is Activated by Hyperpolarization and Regulated by Cytosolic Calcium. Biochem Biophys Res Commun (1999) 261:488–92. doi: 10.1006/bbrc.1999.1059 10425212

[19] van der Hagen EA van Loon EP Verkaart S Latta F Bindels RJ Hoenderop JG. The Na+/Ca2+ Exchanger 1 (NCX1) Variant 3 as the Major Extrusion System in Renal Distal Tubular Transcellular Ca2+-Transport. Nephron (2015) 131:145–52. doi: 10.1159/000440655 26418956

[20] Magyar CE White KE Rojas R Apodaca G Friedman PA. Plasma Membrane Ca2+-ATPase and NCX1 Na+/Ca2+ Exchanger Expression in Distal Convoluted Tubule Cells. Am J Physiol Renal Physiol (2002) 283:F29–40. doi: 10.1152/ajprenal.00252.2000 12060584

[21] Khositseth S Charngkaew K Boonkrai C Somparn P Uawithya P Chomanee N. Hypercalcemia Induces Targeted Autophagic Degradation of Aquaporin-2 at the Onset of Nephrogenic Diabetes Insipidus. Kidney Int (2017) 91:1070–87. doi: 10.1016/j.kint.2016.12.005 28139295

[22] Procino G Mastrofrancesco L Tamma G Lasorsa DR Ranieri M Stringini G. Calcium-Sensing Receptor and Aquaporin 2 Interplay in Hypercalciuria-Associated Renal Concentrating Defect in Humans An *In Vivo* and *In Vitro* Study. PloS One (2012) 7:e33145. doi: 10.1371/journal.pone.0033145 22403735 PMC3293925

[23] Alexander RT Cordat E Chambrey R Dimke H Eladari D. Acidosis and Urinary Calcium Excretion: Insights From Genetic Disorders. J Am Soc Nephrol (2016) 27:3511–20. doi: 10.1681/ASN.2016030305 PMC5118493 27468975

[24] Staruschenko A. Regulation of Transport in the Connecting Tubule and Cortical Collecting Duct. Compr Physiol (2012) 2:1541–84. doi: 10.1002/cphy.c110052 PMC3516049 23227301

[25] Goodman WG Quarles LD. Development and Progression of Secondary Hyperparathyroidism in Chronic Kidney Disease: Lessons From Molecular Genetics. Kidney Int (2008) 74:276–88. doi: 10.1038/sj.ki.5002287 17568787

[26] Hannan FM Kallay E Chang W Brandi ML Thakker RV. The Calcium-Sensing Receptor in Physiology and in Calcitropic and Noncalcitropic Diseases. Nat Rev Endocrinol (2018) 15:33–51. doi: 10.1038/s41574-018-0115-0 30443043 PMC6535143

[27] Olauson H Lindberg K Amin R Sato T Jia T Goetz R. Parathyroid-Specific Deletion of Klotho Unravels a Novel Calcineurin-Dependent FGF23 Signaling Pathway That Regulates PTH Secretion. PloS Genet (2013) 9:e1003975. doi: 10.1371/journal.pgen.1003975 24348262 PMC3861040

[28] Umar M Sastry KS Chouchane AI. Role of Vitamin D Beyond the Skeletal Function: A Review of the Molecular and Clinical Studies. Int J Mol Sci (2018) 19. doi: 10.3390/ijms19061618 PMC6032242 29849001

[29] Poujeol P Chabardes D Roinel N De Rouffignac C. Influence of Extracellular Fluid Volume Expansion on Magnesium, Calcium and Phosphate Handling Along the Rat Nephron. Pflugers Arch (1976) 365:203–11. doi: 10.1007/BF01067020 988560

[30] Dusso AS Brown AJ Slatopolsky E. Vitamin D. Am J Physiol Renal Physiol (2005) 289:F8–28. doi: 10.1152/ajprenal.00336.2004 15951480

[31] Rodriguez-Soriano J Vallo A Castillo G Oliveros R. Natural History of Primary Distal Renal Tubular Acidosis Treated Since Infancy. J Pediatr (1982) 101:669–76. doi: 10.1016/S0022-3476(82)80288-6 7131138

[32] Houillier P Normand M Froissart M Blanchard A Jungers P Paillard M. Calciuric Response to an Acute Acid Load in Healthy Subjects and Hypercalciuric Calcium Stone Formers. Kidney Int (1996) 50:987–97. doi: 10.1038/ki.1996.400 8872975

[33] Lemann J Jr Gray RW Maierhofer WJ Cheung HS. The Importance of Renal Net Acid Excretion as a Determinant of Fasting Urinary Calcium Excretion. Kidney Int (1986) 29:743–6. doi: 10.1038/ki.1986.60 3702225

[34] Lemann J Litzow JR Lennon EJ. Studies of the Mechanism by Which Chronic Metabolic Acidosis Augments Urinary Calcium Excretion in Man. J Clin Invest (1967) 46:1318–28. doi: 10.1172/JCI105624 PMC297133 16695920

[35] Jo MN Seo HJ Kim Y Seo SH Rhim H Cho YS. Novel T-Type Calcium Channel Blockers: Dioxoquinazoline Carboxamide Derivatives. Bioorg Med Chem (2007) 15:365–73. doi: 10.1016/j.bmc.2006.09.051 17035033

[36] Nijenhuis T Renkema KY Hoenderop JG Bindels RJ. Acid-Base Status Determines the Renal Expression of Ca2+ and Mg2+ Transport Proteins. J Am Soc Nephrol (2006) 17:617–26. doi: 10.1681/ASN.2005070732 16421227

[37] Suki WN Martinez-Maldonado M Rouse D Terry A. Effect of Expansion of Extracellular Fluid Volume on Renal Phosphate Handling. J Clin Invest (1969) 48:1888–94. doi: 10.1172/JCI106155 PMC322425 5822594

[38] Alexander RT Dimke H. Effect of Diuretics on Renal Tubular Transport of Calcium and Magnesium. Am J Physiol Renal Physiol (2017) 312:F998–1015. doi: 10.1152/ajprenal.00032.2017 28274923

[39] Kurtz I Star R Balaban RS Garvin JL Knepper MA. Spontaneous Luminal Disequilibrium pH in S3 Proximal Tubules. Role in Ammonia and Bicarbonate Transport. J Clin Invest (1986) 78:989–96. doi: 10.1172/JCI112690 PMC423740 3760195

[40] van Bommel EJ Muskiet MH Tonneijck L Kramer MH Nieuwdorp M van Raalte DH. SGLT2 Inhibition in the Diabetic Kidney-From Mechanisms to Clinical Outcome. Clin J Am Soc Nephrol (2017) 12:700–10. doi: 10.2215/CJN.06080616 PMC5383382 28254770

[41] Haas M Forbush B 3rd. The Na-K-Cl Cotransporters. J Bioenerg Biomembr (1998) 30:161–72. doi: 10.1023/A:1020521308985 9672238

[42] Pacifici GM. Clinical Pharmacology of Furosemide in Neonates: A Review. Pharmaceuticals (Basel) (2013) 6:1094–129. doi: 10.3390/ph6091094 PMC3818833 24276421

[43] Kurtz I. Molecular Pathogenesis of Bartter's and Gitelman's Syndromes. Kidney Int (1998) 54:1396–410. doi: 10.1046/j.1523-1755.1998.00124.x 9767561

[44] Nijenhuis T Vallon V van der Kemp AW Loffing J Hoenderop JG Bindels RJ. Enhanced Passive Ca2+ Reabsorption and Reduced Mg2+ Channel Abundance Explains Thiazide-Induced Hypocalciuria and Hypomagnesemia. J Clin Invest (2005) 115:1651–8. doi: 10.1172/JCI24134 PMC1090474 15902302

[45] Gesek FA Friedman PA. Mechanism of Calcium Transport Stimulated by Chlorothiazide in Mouse Distal Convoluted Tubule Cells. J Clin Invest (1992) 90:429–38. doi: 10.1172/JCI115878 PMC443118 1322939

[46] Shimizu T Nakamura M Yoshitomi K Imai M. Interaction of Trichlormethiazide or Amiloride With PTH in Stimulating Ca2+ Absorption in Rabbit CNT. Am J Physiol (1991) 261:F36–43. doi: 10.1152/ajprenal.1991.261.1.F36 1858902

[47] Friedman PA. Codependence of Renal Calcium and Sodium Transport. Annu Rev Physiol (1998) 60:179–97. doi: 10.1146/annurev.physiol.60.1.179 9558460

[48] Nijenhuis T Hoenderop JG Loffing J van der Kemp AW van Os CH Bindels RJ. Thiazide-Induced Hypocalciuria Is Accompanied by a Decreased Expression of Ca2+ Transport Proteins in Kidney. Kidney Int (2003) 64:555–64. doi: 10.1046/j.1523-1755.2003.00128.x 12846750

[49] Christensson T Hellstrom K Wengle B. Hypercalcemia and Primary Hyperparathyroidism. Prevalence in Patients Receiving Thiazides as Detected in a Health Screen. Arch Intern Med (1977) 137:1138–42. doi: 10.1001/archinte.1977.03630210024009 901082

[50] Friedman PA Gesek FA. Stimulation of Calcium Transport by Amiloride in Mouse Distal Convoluted Tubule Cells. Kidney Int (1995) 48:1427–34. doi: 10.1038/ki.1995.432 8544399

[51] Bhandari S Turney JH. The Molecular Basis of Hypokalaemic Alkalosis: Bartter's and Gitelman's Syndromes. Nephron (1998) 80:373–9. doi: 10.1159/000045209 9832636

[52] Costanzo LS. Localization of Diuretic Action in Microperfused Rat Distal Tubules: Ca and Na Transport. Am J Physiol (1985) 248:F527–35. doi: 10.1152/ajprenal.1985.248.4.F527 3985160

[53] Salcuni AS Palmieri S Carnevale V Morelli V Battista C Guarnieri V. Bone Involvement in Aldosteronism. J Bone Miner Res (2012) 27:2217–22. doi: 10.1002/jbmr.1660 22589146

[54] Krajisnik T Bjorklund P Marsell R Ljunggren O Akerstrom G Jonsson KB. Fibroblast Growth Factor-23 Regulates Parathyroid Hormone and 1alpha-Hydroxylase Expression in Cultured Bovine Parathyroid Cells. J Endocrinol (2007) 195:125–31. doi: 10.1677/JOE-07-0267 17911404

[55] Lee JJ Plain A Beggs MR Dimke H Alexander RT. Effects of Phospho- and Calciotropic Hormones on Electrolyte Transport in the Proximal Tubule. F1000Res (2017) 6:1797. doi: 10.12688/f1000research.12097.1 29043081 PMC5627579

[56] Mace ML Olgaard K Lewin E. New Aspects of the Kidney in the Regulation of Fibroblast Growth Factor 23 (FGF23) and Mineral Homeostasis. Int J Mol Sci (2020) 21. doi: 10.3390/ijms21228810 PMC7699902 33233840

[57] Bikle D Christakos S. New Aspects of Vitamin D Metabolism and Action - Addressing the Skin as Source and Target. Nat Rev Endocrinol (2020) 16:234–52. doi: 10.1038/s41574-019-0312-5 32029884

[58] Levine MA. Diagnosis and Management of Vitamin D Dependent Rickets. Front Pediatr (2020) 8:315. doi: 10.3389/fped.2020.00315 32596195 PMC7303887

[59] Guerboub AA Moussaoui S Issouani J Errahali Y Belmejdoub G. X-Linked Vitamin D-Resistant Rickets: 12 Years of Follow-Up. Pan Afr Med J (2018) 30:9. doi: 10.11604/pamj.2018.30.9.14762 PMC6093588 30123412

[60] Hannan FM Thakker RV. Calcium-Sensing Receptor (CaSR) Mutations and Disorders of Calcium, Electrolyte and Water Metabolism. Best Pract Res Clin Endocrinol Metab (2013) 27:359–71. doi: 10.1016/j.beem.2013.04.007 23856265

[61] Gorvin CM Stokes VJ Boon H Cranston T Gluck AK Bahl S. Activating Mutations of the G-Protein Subunit Alpha 11 Interdomain Interface Cause Autosomal Dominant Hypocalcemia Type 2. J Clin Endocrinol Metab (2020) 105. doi: 10.1210/clinem/dgz251 PMC7048683 31820785

[62] Baumber L Tufarelli C Patel S King P Johnson CA Maher ER. Identification of a Novel Mutation Disrupting the DNA Binding Activity of GCM2 in Autosomal Recessive Familial Isolated Hypoparathyroidism. J Med Genet (2005) 42:443–8. doi: 10.1136/jmg.2004.026898 PMC1736051 15863676

[63] Gordon RJ Levine MA. Genetic Disorders of Parathyroid Development and Function. Endocrinol Metab Clin North Am (2018) 47:809–23. doi: 10.1016/j.ecl.2018.07.007 PMC6233988 30390815

[64] Marx SJ Lourenco DM Jr. Familial Hyperparathyroidism - Disorders of Growth and Secretion in Hormone-Secretory Tissue. Horm Metab Res (2017) 49:805–15. doi: 10.1055/s-0043-120670 29136674

[65] Wilson M Carter IB. Williams Syndrome. In: StatPearls (2021). 31334998

[66] Karmiloff-Smith A Broadbent H Farran EK Longhi E D'Souza D Metcalfe K. Social Cognition in Williams Syndrome: Genotype/Phenotype Insights From Partial Deletion Patients. Front Psychol (2012) 3:168. doi: 10.3389/fpsyg.2012.00168 22661963 PMC3362742

[67] Mrad FCC Soares SBM de Menezes Silva LAW Dos Anjos Menezes PV Simoes ESAC. Bartter's Syndrome: Clinical Findings, Genetic Causes and Therapeutic Approach. World J Pediatr (2021) 17:31–9. doi: 10.1007/s12519-020-00370-4 32488762

[68] Komholf M Laghmani K. MAGED2 a Neovel Form of Antenatal Bartter's Syndrome. Curr Opin Nephrol Hypertens (2018) 27:323–28. 10.1097/MNH.0000000000000422 29677005

[69] Besnouw M Besou. Bartter and Gitelman syndrome:Question of Class. Pediatr Nephrol (2020) 27(4):323–8. doi: 10.1097/MNH.0000000000000422

[70] Nakhoul F Nakhoul N Dorman E Berger L Skorecki K Magen D. Gitelman's Syndrome: A Pathophysiological and Clinical Update. Endocrine (2012) 41:53–7. doi: 10.1007/s12020-011-9556-0 22169961

[71] Mabillard H Sayer JA. The Molecular Genetics of Gordon Syndrome. Genes (Basel) (2019) 10. doi: 10.3390/genes10120986 PMC6947027 31795491

[72] Celmina M Micule I Inashkina I Audere M Kuske S Pereca J. EAST/SeSAME Syndrome: Review of the Literature and Introduction of Four New Latvian Patients. Clin Genet (2019) 95:63–78. doi: 10.1111/cge.13374 29722015

[73] Abdelhadi O Iancu D Stanescu H Kleta R Bockenhauer D. EAST Syndrome: Clinical, Pathophysiological, and Genetic Aspects of Mutations in KCNJ10. Rare Dis (2016) 4:e1195043. doi: 10.1080/21675511.2016.1195043 27500072 PMC4961265

[74] Hampson G Konrad MA Scoble J. Familial Hypomagnesaemia With Hypercalciuria and Nephrocalcinosis (FHHNC): Compound Heterozygous Mutation in the Claudin 16 (CLDN16) Gene. BMC Nephrol (2008) 9:12. doi: 10.1186/1471-2369-9-12 18816383 PMC2562370

