# Supplementary material for: Calcium Transport in the Kidney and Disease Processes
Source: Front Endocrinol (Lausanne). 2022 Mar 1;12:762130. doi: 10.3389/fendo.2021.762130 (PMC8922474; doi:10.3389/fendo.2021.762130)
Supplement: Supplemental Figure A — Nephron Phosphate Handling: CNI, calcineurin pathway; FGF23, fibroblast growth factor 23; FGF23R, fibroblast growth factor receptor; HPO4 2-, H2PO4 -, organic phosphate anions; MAPK, map kinase; NHERF-1, Na+/H+ exchanger regulatory factor 1; Na K ATPase, sodium potassium ATPase; NaPi2a, sodium phosphate cotransporter 2a; NaPi2c, sodium phosphate cotransporter 2c; PiT2, phosphate transporter; PKA/PKC, protein kinase A/C; PTH, parathyroid hormone. [file Presentation_1.pptx]

## Slide 1
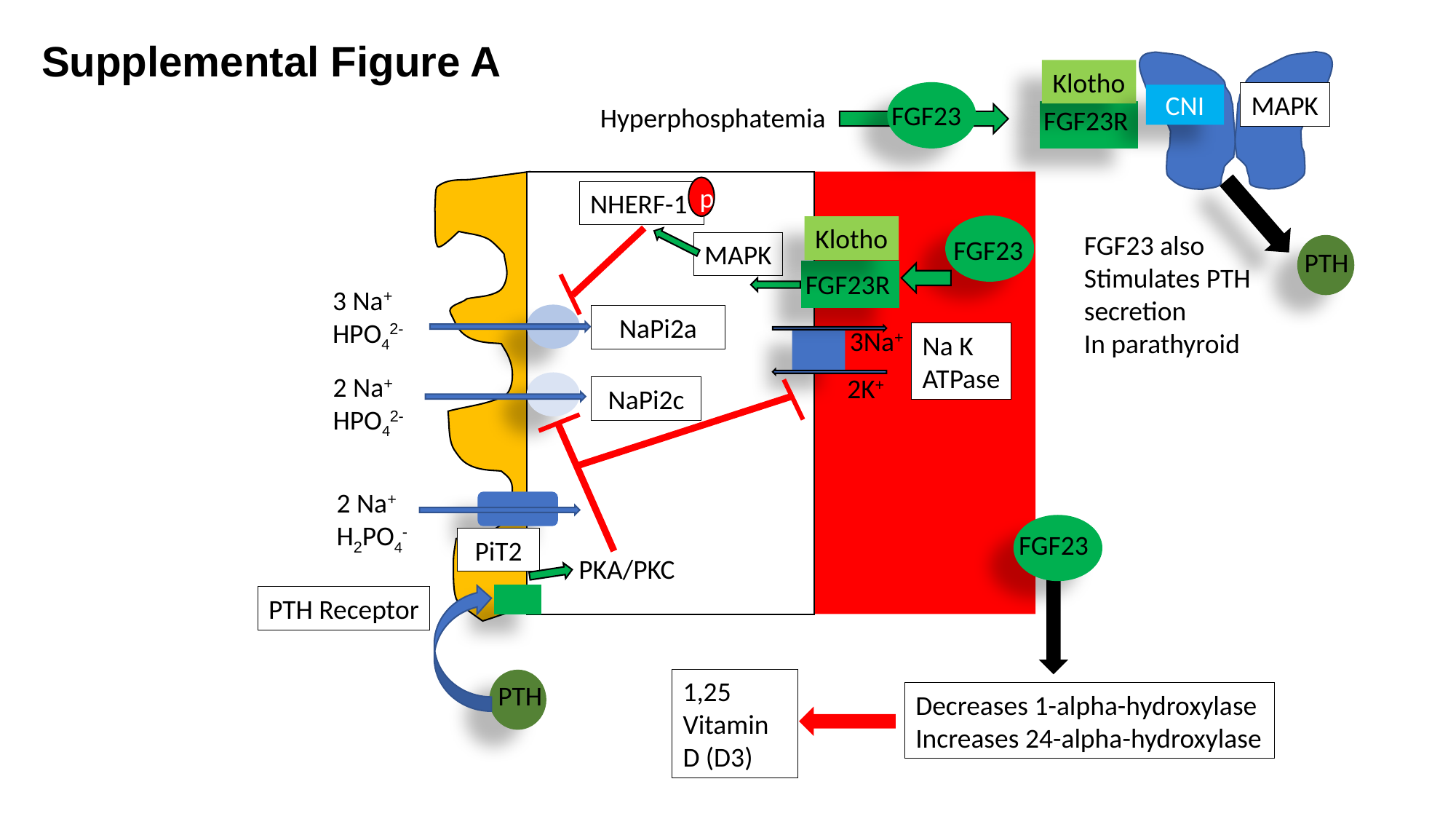

Supplemental Figure A
Klotho
MAPK
CNI
FGF23
Hyperphosphatemia
FGF23R
p
NHERF-1
Klotho
FGF23 also
Stimulates PTH
secretion
In parathyroid
FGF23
MAPK
PTH
FGF23R
3 Na+
HPO42-
NaPi2a
3Na+
Na K
ATPase
2 Na+
HPO42-
2K+
NaPi2c
2 Na+
H2PO4-
FGF23
PiT2
PKA/PKC
PTH Receptor
1,25 Vitamin D (D3)
PTH
Decreases 1-alpha-hydroxylase
Increases 24-alpha-hydroxylase
