# Supplementary material for: Calcium Transport in the Kidney and Disease Processes
Source: Front Endocrinol (Lausanne). 2022 Mar 1;12:762130. doi: 10.3389/fendo.2021.762130 (PMC8922474; doi:10.3389/fendo.2021.762130)
Supplement: Supplementary file 2 [file Table_1.docx]

**Supplemental Table A: Presentation of Common Genetic Syndromes Affecting Calcium Metabolism**

| Syndrome | Serum Calcium | Serum  Potassium | Serum  Magnesium | Urine Calcium | Urine  Potassium | Urine Magnesium | Blood  Pressure | Volume |
| --- | --- | --- | --- | --- | --- | --- | --- | --- |
| Bartter’s Syndrome | Normal | Low | Low- Normal | Normal-High | High | High | Low-Normal | Hypovolemic to Euvolemic |
| Gitelman’s  Syndrome | Normal | Low | Low-  Very Low | Low | High | High-  Very High | Low-Normal | Hypovolemic to Euvolemic |
| Gordon’s Syndrome | Normal | High | Normal | High | Low | Normal | High | Euvolemic to Hypervolemic |
| William’s  Syndrome | High | Normal | Normal | High | Normal | Normal | High | Hypovolemic to Euvolemic |
| EAST/Sesame  Syndrome | Normal | Low | Low-  Very Low | Low | High | High-  Very High | Low-Normal | Hypovolemic to Euvolemic |
| FHHNC  Syndrome | Normal-High | Normal | Normal | High | Normal | Normal | Normal | Euvolemic |
